# Cortical Excitability as a Prognostic and Phenotypic Stratification Biomarker in Amyotrophic Lateral Sclerosis

**DOI:** 10.1002/ana.27305

**Published:** 2025-06-25

**Authors:** Federico Ranieri, Gianmaria Senerchia, Luigi Bonan, Stefania Casali, Corrado Cabona, Mariagiovanna Cantone, Fabiola De Marchi, Luca Diamanti, Alberto Doretti, Nicola Fini, Massimiliano Filosto, Andrea Fortuna, Aniello Iovino, Valentina Virginia Iuzzolino, Giuseppe Lanza, Christian Lunetta, Luca Maderna, Jessica Mandrioli, Letizia Mazzini, Gabriella Musumeci, Andi Nuredini, Gianni Sorarù, Antonella Toriello, Nicola Ticozzi, Massimiliano Todisco, Veria Vacchiano, Lucia Zinno, Vincenzo Silani, Simone Rossi, Vincenzo Di Lazzaro, Raffaele Dubbioso, the MND Study Group of the Italian Neurological Society (SIN) and the Neurostimulation‐Neuromodulation Study Group of the Italian Society of Clinical Neurophysiology (SINC), Francesca Calvi, Laura Filippi, Marilisa Boscarino, Rita Bella, Maria Caputo, Stefano Gazzina, Rachele Piras, Manuela Pennisi, Fabio Pilato, Rosa Iodice

**Affiliations:** ^1^ Neurology Unit, Department of Neuroscience, Biomedicine, and Movement Sciences University of Verona Verona Italy; ^2^ Department of Neurosciences, Reproductive Sciences, and Odontostomatology University of Naples Federico II Naples Italy; ^3^ Department of Biomedical and Neuromotor Sciences University of Bologna Bologna Italy; ^4^ Unit of Neurology and Clinical Neurophysiology, Department of Medicine, Surgery, and Neuroscience University of Siena Siena Italy; ^5^ Division of Clinical Neurophysiology, IRCCS Ospedale Policlinico San Martino Genoa Italy; ^6^ Neurology Unit Policlinico University Hospital “G. Rodolico‐San Marco” Catania Italy; ^7^ ALS Center “Maggiore della Carità” University Hospital, University of Piemonte Orientale Novara Italy; ^8^ Neuro‐Oncology Unit IRCCS Mondino Foundation Pavia Italy; ^9^ Department of Neurology and Laboratory of Neuroscience IRCCS Istituto Auxologico Italiano Milan Italy; ^10^ Department of Neurosciences Azienda Ospedaliero Universitaria di Modena Modena Italy; ^11^ NeMO‐Brescia Clinical Center for Neuromuscular Diseases ERN Euro‐NMD Center ASST Spedali Civili Brescia Italy; ^12^ Department of Clinical and Experimental Sciences University of Brescia Brescia Italy; ^13^ Veneto Regional Center Motor Neuron Diseases, Department of Neurosciences University Hospital of Padova Padova Italy; ^14^ Neurology Unit, University Hospital “San Giovanni di Dio e Ruggi d'Aragona” University of Salerno Salerno Italy; ^15^ Clinical Neurophysiology Research Unit Oasi Research Institute‐IRCCS Troina Italy; ^16^ Department of Surgery and Medical‐Surgical Specialties University of Catania Catania Italy; ^17^ Istituti Clinici Scientifici Maugeri IRCCS, Neurorehabilitation Department, Institute of Milan Milan Italy; ^18^ Department of Biomedical, Metabolic and Neural Sciences University of Modena and Reggio Emilia Modena Italy; ^19^ Department of Medicine and Surgery, Unit of Neurology, Neurophysiology, Neurobiology, and Psychiatry Università Campus Bio‐Medico di Roma Roma Italy; ^20^ Department of Medicine and Surgery University of Parma Parma Italy; ^21^ Department of Pathophysiology and Transplantation, Dino Ferrari Center Università degli Studi di Milano Milan Italy; ^22^ Neurophysiology Unit IRCCS Mondino Foundation Pavia Italy; ^23^ Siena Brain Investigation & Neuromodulation Lab (Si‐BIN Lab), Unit of Neurology and Clinical Neurophysiology, Department of Medicine, Surgery, and Neuroscience University of Siena Siena Italy; ^24^ Fondazione Policlinico Universitario Campus Bio‐medico, Via Alvaro del Portillo Rome Italy

## Abstract

**Objective:**

Despite its clinical heterogeneity, amyotrophic lateral sclerosis is unified by early and prominent alterations in cortical excitability, increasingly recognized as contributors to disease progression. This study assessed whether the ratio between motor evoked potential (MEP) amplitude, reflecting upper motor neuron integrity, and compound muscle action potential (CMAP) amplitude, indexing lower motor neuron function, could provide an accessible marker of corticospinal excitability to stratify patients by phenotype, stage, and survival.

**Methods:**

In this multicenter retrospective study, 743 amyotrophic lateral sclerosis patients from 16 tertiary centers in Italy were analyzed. The MEP:CMAP ratio, recorded from upper limb muscles, was categorized as hyperexcitable, normal, or hypoexcitable. Phenotypes included progressive muscular atrophy (or lower motor neuron), flail arm/leg, classic, bulbar, patient with predominant upper motor neuron signs (or pyramidal), and primary lateral sclerosis. Disease stage was assessed using King's staging. Survival was analyzed using Kaplan–Meier curves and Cox regression models.

**Results:**

The MEP:CMAP ratio differed significantly across phenotypes (*p* < 0.0001), with hyperexcitability predominating in lower motor neuron, flail, classic, and bulbar forms, and hypoexcitability in pyramidal and primary lateral sclerosis. Hypoexcitability increased in advanced King's stages (*p* < 0.0001). Hyperexcitable patients had shorter survival (*p* = 0.004), including when tested within 1 year of onset (*p* = 0.006). Cox regression identified the MEP:CMAP ratio as an independent survival predictor (HR 1.84, 95% CI 1.12–3.03, *p* = 0.016).

**Interpretation:**

This real‐world study supports the clinical value of the MEP:CMAP ratio as a scalable biomarker of cortical excitability in amyotrophic lateral sclerosis, with prognostic relevance across phenotypes and disease stages. ANN NEUROL 2025;98:801–813

Amyotrophic lateral sclerosis (ALS) is a progressive neurodegenerative disease characterized by the degeneration of upper motor neurons (UMNs) and lower motor neurons (LMNs), resulting in severe motor disability and, ultimately, death from respiratory failure.^1^ Despite its well‐documented clinical heterogeneity, ALS is unified by a key pathophysiological hallmark: altered cortical excitability, which is increasingly recognized as a critical driver of disease onset and progression.^2^, ^3^, ^4^, ^5^, ^6^

Cortical hyperexcitability has been identified as an early feature of ALS, preceding the onset of LMN dysfunction^7^ and the appearance of motor symptoms in pre‐symptomatic familial cases.^8^ This phenomenon is thought to result from an imbalance between excitatory and inhibitory mechanisms in the motor cortex, with impaired GABAergic inhibition and increased glutamatergic activity driving excitotoxic damage to motor neurons.^9^, ^10^, ^11^ Hyperexcitability, reflected by reduced short‐interval intracortical inhibition at paired‐pulse transcranial magnetic stimulation (TMS) paradigms, is particularly pronounced in the early stages of ALS (<24 months), and strongly correlates with reduced survival.^12^

Interestingly, cortical excitability differs across ALS phenotypes. Patients with classic ALS show marked hyperexcitability, as also observed in those with flail arm or flail leg variants, despite the reduced number of available motor units due to predominant LMN degeneration.^7^, ^13^ In contrast, cortical inexcitability, defined as the absence of motor evoked potentials (MEPs), even at maximal single pulse TMS intensity, reflects advanced corticospinal degeneration.^14^ This state, associated with younger age at onset, advanced disease stage, and higher UMN clinical burden,^15^ highlights the heterogeneity of excitability patterns across ALS phenotypes, where hypoexcitability and inexcitability coexist with hyperexcitability depending on disease stage and phenotype.^15^

This heterogeneity of cortical excitability underscores the critical need for reliable markers of UMN involvement, as current ALS diagnostic criteria still rely solely on clinical assessments.^16^, ^17^

From a neurophysiological perspective, combining MEP amplitude, which reflects UMN integrity, with compound muscle action potential (CMAP) amplitude, representing the number of preserved LMN axons, yields a ratio that provides a routinely clinically accessible measure of corticospinal excitability, normalized to peripheral motor function.^18^ Thus, the MEP:CMAP ratio could offer insights into cortical excitability while accounting for the bias introduced by motor units degeneration.^19^ Unlike paired‐pulse TMS^20^ and other more complex techniques, such as threshold‐tracking TMS,^21^, ^22^ this biomarker offers a practical and scalable approach, enabling the assessment of excitability along a continuum from hyperexcitability to inexcitability. This simplicity makes the MEP:CMAP ratio particularly valuable for routine clinical use.

The present study aimed to evaluate the clinical utility of the MEP:CMAP ratio in ALS, with a particular focus on its ability to stratify patients across different phenotypes and disease stages. This approach seeks to comprehensively explore the entire spectrum of cortical excitability, and its impact on survival and functional outcomes.

Although paired‐pulse and threshold‐tracking TMS techniques provide valuable insights into cortical excitability, particularly within normal and hyperexcitable ranges, they offer limited information when corticospinal output becomes markedly reduced or absent, as these methods require measurable responses to function. In contrast, the MEP:CMAP ratio captures a broader range of corticospinal states, including hypoexcitable and inexcitable conditions, thus complementing conventional TMS measures by allowing classification of patients with absent or markedly reduced cortical responses, who may otherwise remain underrepresented.

By delineating the transition from early hyperexcitability to late hypoexcitability and cortical inexcitability, this study advances our understanding of ALS pathophysiology, and underscores the potential of excitability biomarkers, such as the MEP:CMAP ratio, to enhance patient stratification, guide therapeutic strategies, and support the development of targeted interventions to modulate cortical dysfunction and mitigate excitotoxicity.

## Materials and Methods

### Patients

**FIGURE 1:**
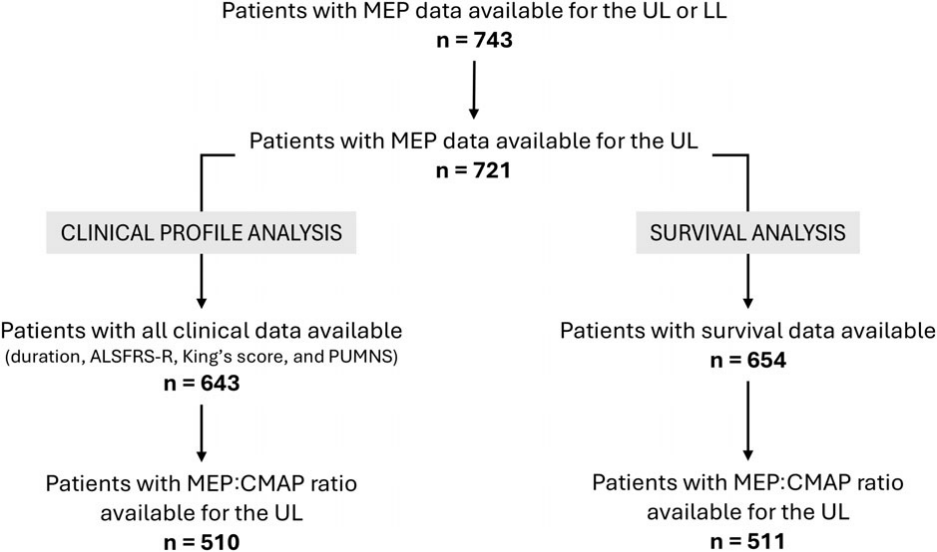
Flowchart of patient selection from a large cohort who underwent motor evoked potential examination for diagnostic purposes. ALSFRS‐R = Amyotrophic Lateral Sclerosis Functional Rating Scale Revised; CMAP = compound muscle action potential; LL = lower limb; MEP = motor evoked potential; PUMNS = Penn upper motor neuron score; UL = upper limb.

This retrospective analysis included MEP assessments from 743 patients who underwent electrophysiological testing for diagnostic purposes. These data were gathered from 16 different tertiary referral centers across Italy between 2002 and 2023. Among these, 721 patients had upper limb MEP measurements, and 643 patients also had a full set of clinical data available (Fig 1). ALS patients met the El Escorial criteria,^23^ which include cases classified as “suspected”, “possible”, “probable”, “probable laboratory‐supported,” and “definite ALS”. As this was a retrospective cohort, diagnoses were subsequently confirmed during follow up based on clinical progression. Primary lateral sclerosis (PLS) patients were diagnosed based on the criteria outlined by Turner et al.^24^

Demographic data, including age and sex, were recorded for all participants. The time from disease onset to the TMS study, which corresponded to the clinical evaluation, was calculated in months to assess disease duration. The site of symptom onset was categorized as “spinal,” “bulbar,” or “unknown.” In addition, patients were classified into clinical phenotypes, including classic ALS (with limb onset and both UMN and LMN signs without clear predominance), bulbar phenotype (with bulbar onset and both UMN and LMN signs), flail phenotypes (flail arm and flail leg, characterized by regional LMN weakness and wasting, with mild UMN signs, such as brisk reflexes), pyramidal phenotype (predominant UMN signs with mild LMN involvement), pure lower motor neuron phenotype or progressive muscular atrophy (progressive LMN involvement without UMN signs), and PLS (exclusive and progressive UMN involvement without LMN signs).^25^

Functional status was evaluated using the ALS Functional Rating Scale‐Revised (ALSFRS‐R), with scores ranging from 0 (worst) to 48 (best), reflecting the extent of functional impairment.^26^ Disease severity was assessed using the King's clinical staging system, which evaluates anatomical involvement in the bulbar, upper limb, and lower limb regions.^27^ Considering the limited number of patients in stage 4 (*n* = 50), and the intent to evaluate the spreading of the disease across the 3 body regions, we restricted the analysis to stages 1 to 3. Nevertheless, patients in stage 4 were reclassified into stages 1 to 3 according to the extent of regional involvement.

The progression of functional decline was quantified using the ALSFRS‐R monthly progression rate (ALSFRS‐R rate), calculated as the difference between 48 (full score) and the ALSFRS‐R score at evaluation, divided by the time (in months) from symptom onset to evaluation.^28^ UMN involvement was quantified using the Penn Upper Motor Neuron Score (PUMNS), which evaluates bulbar, upper limb, and lower limb regions and provides subscores for each.^29^ For this retrospective analysis, PUMNS was reconstructed based on clinical records available at the time of neurophysiological assessment. Given that most participating centers are specialized ALS referral clinics, reflex examination was routinely performed and recorded. When specific signs were missing from the records, the corresponding items were considered not assessable and excluded from the subscore calculation. Overall, data were available for 643 patients (Fig 1).

Genetic analysis was performed in a subgroup of patients to identify pathogenic mutations. Data on key genes, including *C9ORF72, SOD1*, *TARDBP*, and *FUS*, were collected. Patients without identifiable mutations were categorized as non‐mutated, whereas others were classified based on specific genetic variants detected.

The study protocol was approved by the local Ethical Committees of the participating centers, and carried out in accordance with the Declaration of Helsinki.

### Neurophysiological Assessment

TMS was applied using a 90‐mm circular coil connected to a magnetic stimulator: Magstim models, *n* = 480 patients (Magstim, Whitland, UK); Ates STM9000, *n* = 173 (EB Neuro SpA, Firenze, Italy); and MagPro models, *n* = 90 (MagVenture, Farum, Denmark). The coil was positioned over the vertex, with its placement adjusted to evoke the largest MEP response from the target muscles. Based on central motor conduction time (CMCT) measurement, the participating centers were asked to provide MEP data from the most pathological side (ie, higher CMCT value or absent cortical response) at a minimum, or from the dominant side in cases of normal findings. Neurophysiological parameters were evaluated from 3 hand muscles, including the first dorsal interosseous, other thenar muscles (ie, abductor pollicis brevis or opponens pollicis), or abductor digiti minimi muscles, and aggregate values were reported for statistical analysis.

The MEP:CMAP ratio was calculated to normalize variability in peripheral responses and provide an accurate measure of corticospinal excitability. Values >1, typically due to contributions from surrounding muscles, were capped at 1 for analysis. CMCT was calculated by subtracting the estimated peripheral conduction time, obtained after root stimulation, from the total MEP latency. CMCT data were categorized into 4 groups: normal findings (N), pathological slowing of CMCT (P), non‐evocable cortical response (NEC) with preserved peripheral response, and non‐evocable peripheral response (NEP). Pathological slowing of CMCT was defined based on the normative data of each neurophysiological laboratory, with thresholds set at least 2 standard deviations above the expected mean. All measurements were performed in accordance with updated guidelines for TMS use in clinical practice and research applications.^19^, ^30^, ^31^

Cortical excitability was categorized into 3 states—hypoexcitable, normal, and hyperexcitable—based on thresholds derived from a convenience sample of healthy controls recruited at the Federico II University Hospital of Naples (Naples, Italy). Further methodological details, including stimulation parameters, classification criteria, and control thresholds, are provided in S1.

### Statistical Analysis

All statistical analyses were conducted using SPSS (IBM SPSS Statistics for Windows, Version 29.0; IBM Corporation, Armonk, NY, USA). As the data were not normally distributed, as determined by the Shapiro–Wilk test, nonparametric statistical methods were applied. Continuous variables were presented as medians with interquartile ranges (IQR), and analyzed using the Mann–Whitney *U* test for 2‐group comparisons or the Kruskal–Wallis test for multiple groups. Comparisons between categorical variables were performed using the χ^2^ test, with pairwise post‐hoc comparisons for Kruskal–Wallis and proportions adjusted via the Bonferroni method.

Survival, defined as the time from TMS testing to death or last follow up for censored cases, was analyzed using Kaplan–Meier survival curves and Cox proportional hazards regression models. Kaplan–Meier curves were used to compare survival between the 2 pathological cortical excitability states—hypoexcitable and hyperexcitable—with statistical differences assessed using the log‐rank test. Cox proportional hazards regression analysis was performed to evaluate the prognostic value of clinical and neurophysiological variables. Clinical variables (age, sex, riluzole use, ALSFRS‐R rate, onset site, phenotype group, and PUMNS total score) were entered a priori based on their established relevance.^26^ Neurophysiological variables (MEP:CMAP ratio and CMCT) were subsequently evaluated for inclusion using a stepwise forward likelihood ratio approach to identify additional predictors of survival while minimizing model complexity. Results are reported as hazard ratios (HRs) with 95% confidence intervals (CIs). All graphs and visualizations were created using R (version 4.4.2; The R Foundation for Statistical Reporting, Vienna, Austria) and GraphPad Prism (version 8.4.2; San Diego, CA, USA). Statistical significance was set at *p* < 0.05, with adjusted *p* values reported for multiple comparisons after Bonferroni correction to control for type I error.

## Results

### Clinical, Demographic, and Genetic Data of ALS Patients

A total of 721 ALS patients were included in this study. The cohort were predominantly men (*n* = 426, 59.1%). The median age at the time of TMS study was 62.6 years (IQR 55–71 years). On average, patients were assessed 19.2 months after symptom onset (IQR 8–23 months).

Regarding the site of onset, most patients presented with spinal onset (*n* = 546, 75.7%), followed by bulbar onset (*n* = 165, 22.9%). A small proportion (*n* = 10, 1.4%) had an unknown onset site. Phenotypic analysis revealed that the classic spinal was the most common (*n* = 359, 49.8%), followed by bulbar forms (*n* = 107, 14.8%), PLS (*n* = 81, 11.2%), the flail phenotypes (*n* = 68, 9.4%), pyramidal ALS (*n* = 59, 8.2%), and the LMN variant (*n* = 47, 6.5%).

King's staging, available in 690 patients of the cohort, was used to evaluate the functional anatomical involvement. Bulbar involvement was present in 58.1% (*n* = 401), upper limb in 63.5% (*n* = 438), and lower limb in 72.8% (*n* = 502). Multidistrict involvement was common, with 32.5% of the patients (*n* = 235) presenting with all 3 districts (bulbar, upper limbs, and lower limbs) affected. Isolated involvement of specific regions was less frequent, with 10.4% (*n* = 72) showing only bulbar involvement, 7.8% (*n* = 54) upper limb involvement, and 17.4% (*n* = 120) lower limb involvement.

Upper motor neuron burden was evaluated across 3 anatomical regions using the PUMNS, which was available in 643 patients of the cohort. The lower limbs were the most frequently affected (*n* = 532, 82.7%), with a median severity score of 5.0 (IQR 2.0–8.0). The upper limbs were affected in 506 patients (78.7%), with a median severity score of 4.0 (IQR 2.0–7.5). Finally, the bulbar region was involved in 356 patients (55.4%), with a median severity score of 1.0 (IQR 0.0–2.0).

Among patients with available genetic data (*n* = 179 out of the entire cohort, excluding those not tested), the most common finding was the absence of pathogenic mutations (*n* = 111, 62.0%). Patients carrying the pathogenic expansions in the *C9ORF72* gene represented the most frequently identified variant, observed in 14.5% of cases (*n* = 26). Other less common mutations included *SOD1* (*n* = 6, 3.4%), *FUS* (*n* = 5, 2.8%), and *TARDBP* (*n* = 5, 2.8%). Additionally, several rare genetic variants were identified in single patients (n = 26, 14.5%).

Treatment data showed that 72.1% of the patients (*n* = 520) were receiving riluzole, 25.7% (*n* = 185) were untreated, and 0.1% (*n* = 1) received edaravone. Treatment status was unknown in 2.1% (*n* = 15).

The observation period spanned from September 2002 to June 2023, with a median survival from the time of observation of 19.0 months (IQR 7.5–30.0 months), among deceased patients. As for the outcomes, 35.6% (*n* = 158/444) used noninvasive ventilation, 11.8% (*n* = 52/439) underwent a tracheostomy, 21.4% (*n* = 93/435) received percutaneous endoscopic gastrostomy, and 39.4% (*n* = 258/654) had died by the latest follow up.

### Neurophysiological Results

MEP and CMAP data across different hand muscles, along with total median values, are presented in Table 1. The proportion of normal central motor conduction time (CMCT) findings was uniform across muscles, comprising 61.4% of the cohort. Pathological findings included P (28.5%) or NEC (8.3%). NEP was infrequent (1.8%). CMCT was slightly longer in thenar muscles with a trend of significance (*p* = 0.07), with values ranging from 7.4 ms (thenar) to 7.0 ms (first dorsal interosseous).

**TABLE 1:** Comparison of Neurophysiological Parameters Across Hand Muscles

	Thenar^a^	ADM	FDI	*p* value	Total
CMCT alteration					
Normal, n (%)	176 (65.7%)	142 (58.0%)	120 (60.0%)	0.178^b^	438 (61.4%)
Pathological, n (%)	63 (23.5%)	80 (32.6%)	60 (30.0%)	0.062^b^	203 (28.5%)
NEC, n (%)	24 (9.0%)	20 (8.2%)	15 (7.5%)	0.85^b^	59 (8.3%)
NEP, n (%)	5 (1.9%)	3 (1.2%)	5 (2.5%)	0.605^b^	13 (1.8%)
Median CMCT value, ms (IQR)	7.40 (6.40–9.80) [*n* = 183]	7.15 (6.30–9.82) [*n* = 166]	7.00 (6.10–9.00) [*n* = 180]	0.074^c^	7.20 (6.29–9.50) [*n* = 529]
Median MEP:CMAP ratio (IQR)	0.32 (0.08–0.70) [*n* = 182]	0.31 (0.12–0.55) [*n* = 200]	0.36 (0.08–0.56) [*n* = 163]	0.788^c^	0.33 (0.10–0.58) [*n* = 545]
Median CMAP amplitude, mV (IQR)	6.50 (3.42–10.65) [*n* = 182]	8.60 (5.47–12.00) [*n* = 200]	8.10 (3.00–13.05) [*n* = 163]	**0.019** ^c^	7.90 (3.90–11.90) [*n* = 545]

^a^ Thenar muscles include abductor pollicis brevis and opponens pollicis muscles. Note: values are expressed as median (interquartile range) or as percentage (frequencies).

^b^ Comparisons χ^2^‐test.

^c^ Kruskal–Wallis test. Values in bold type indicate significance *p* < 0.05.

Abbreviations: ADM = abductor digiti minimi; CMCT = central motor conduction time; CMAP = compound muscle action potential; MEP = motor evoked potential; NEC = non‐evocable cortical response; NEP = non‐evocable peripheral response by cervical stimulation.

**FIGURE 2:**
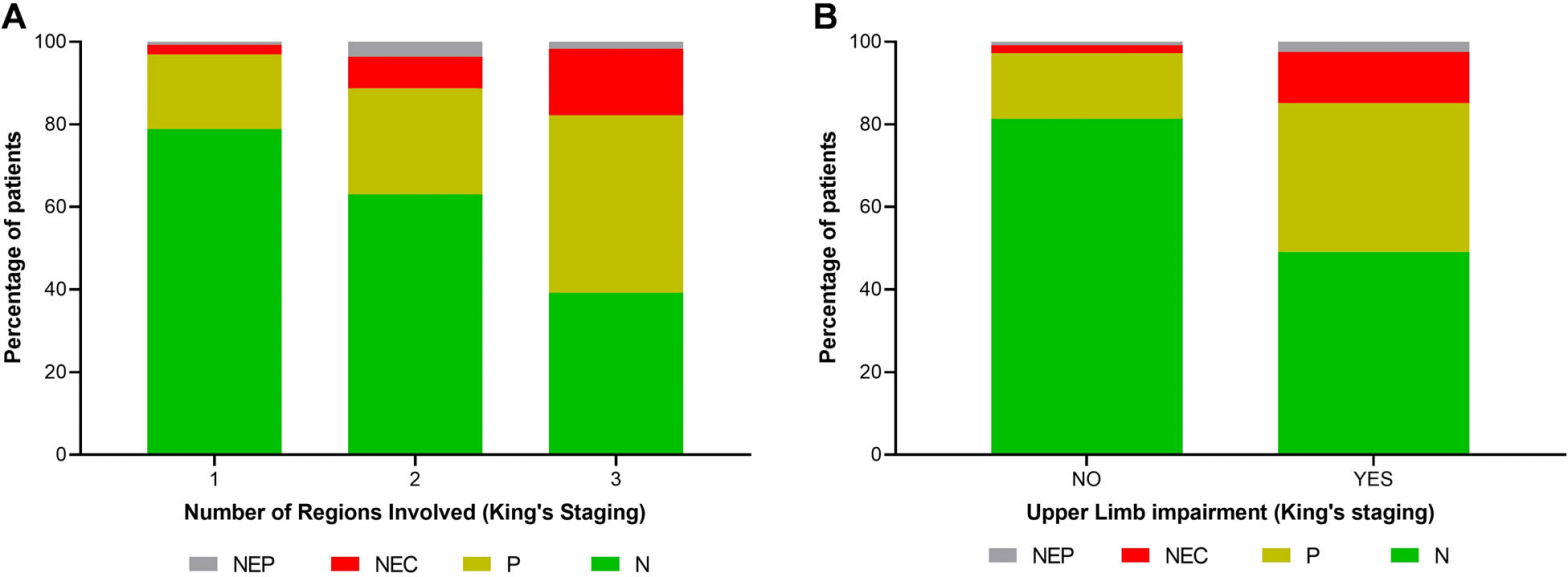
Progression of central motor conduction time (CMCT) alterations in relation to affected body regions and upper limb involvement. (A) Distribution of central motor conduction time is shown across the number of affected body regions (1–3) and (B) according to upper limb regional involvement at King's staging, defined by any reduction in Amyotrophic Lateral Sclerosis Functional Rating Scale‐Revised handwriting (item 4) or cutting food and handling utensils (item 5A) scores. The data indicate that pathological findings become increasingly prevalent both with disease stages (ie, from King's stage 1–3) and with upper limb involvement, in terms of either prolonged CMCT or corticospinal inexcitability (pathological findings, χ^2^ < 0.0001 for both conditions). N = normal CMCT; P = prolonged CMCT; NEC = non‐evocable cortical response (with preserved peripheral response); NEP = non‐evocable peripheral response. [Color figure can be viewed at www.annalsofneurology.org]

Across King's stages, the proportion of pathological findings (ie, merged P, NEC, and NEP) increased significantly with disease severity (χ^2(2,n=690)^ = 88.22, *p* < 0.0001). Pathological findings were observed in 21.2% of stage 1 cases, increasing to 36.9% in stage 2, and 60.9% in Stage 3 (Fig 2). Furthermore, when patients were stratified according to upper limb regional involvement, defined for King's staging as any reduction in handwriting (ALSFRS‐R item 4) or cutting food and handling utensils (item 5A) scores,^32^ a similar pattern emerged in the distribution of pathological CMCT findings relative to disease severity. Specifically, the combined prevalence of P, NEC, and NEP alterations was significantly higher in patients with upper limb impairment compared with those without (50.9% vs 18.7%, χ^2(1,n=690)^ = 69.90, *p* < 0.0001; Fig 2B). Finally, the MEP:CMAP ratio showed no significant differences among muscles (*p* = 0.79), with an overall median value of 0.33 (IQR 0.10–0.58; Table 1).

### Cortical Excitability Profiles by Clinical Phenotype and King's Stages

**FIGURE 3:**
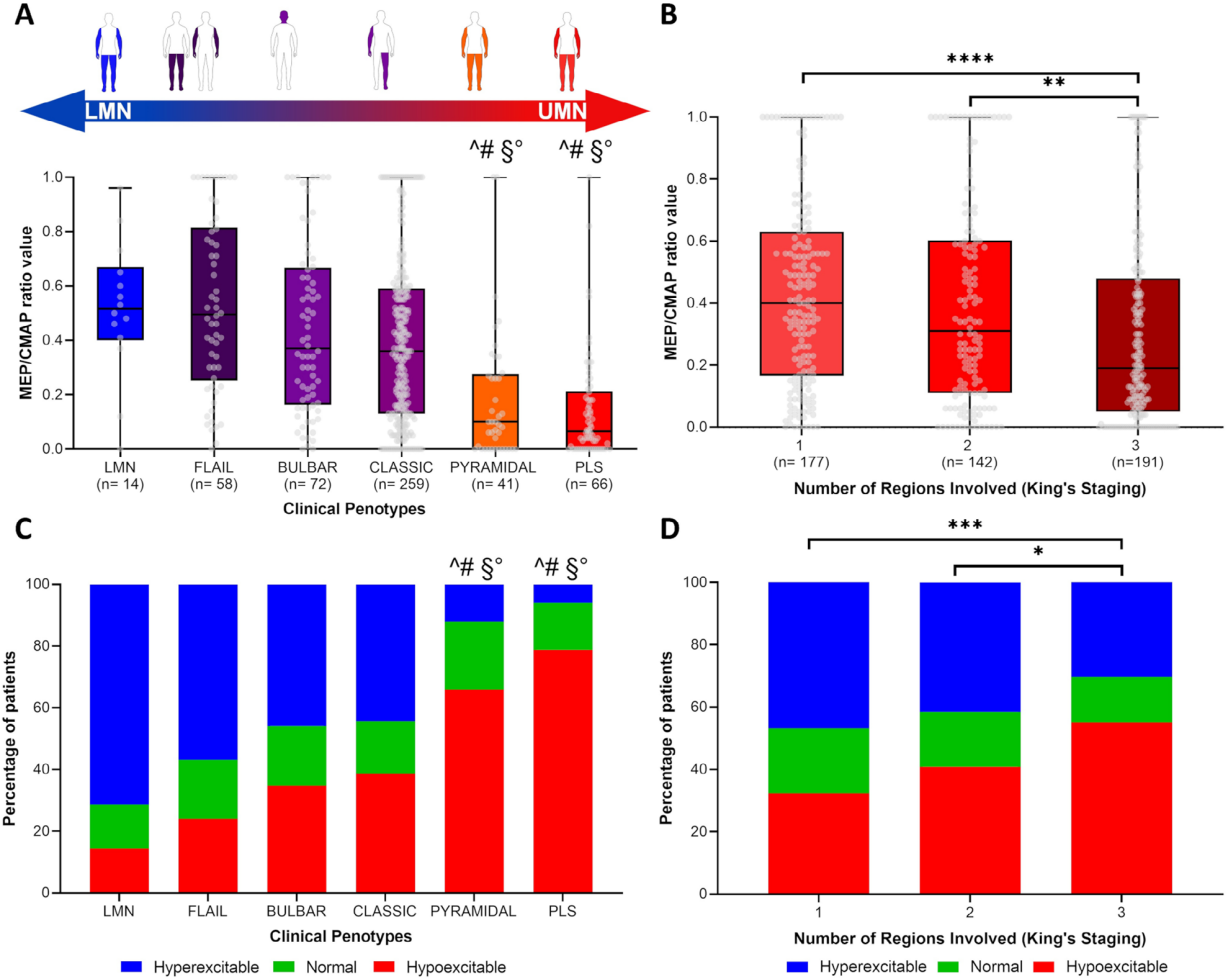
Cortical excitability spectrum across region spreading and phenotypes in amyotrophic lateral sclerosis (ALS). (A) the motor evoked potential/compound muscle action potential (MEP:CMAP) ratio values are shown across ALS phenotypes, ranging from lower motor neuron phenotypes (eg, lower motor neuron [LMN] and flail) to upper motor neuron phenotypes (eg, pyramidal and primary lateral sclerosis [PLS]). The data reveal a significant gradient, with higher MEP:CMAP ratios observed in LMN‐dominant phenotypes, and progressively lower ratios in upper motor neuron (UMN)‐dominant phenotypes (Kruskal–Wallis test, *p* < 0.001). This pattern underscores the increasing burden of corticospinal tract degeneration and the reduction in cortical excitability associated with phenotypes predominantly characterized by UMN dysfunction. (B) MEP:CMAP ratio values are presented across anatomical regions classified by King's staging (1–3). The data demonstrate a significant decline in MEP:CMAP ratios as the number of anatomical regions involved increases (Kruskal–Wallis test, *p* < 0.001). This decline reflects the cumulative axonal degeneration of corticospinal tracts and a corresponding reduction in cortical excitability, which becomes more pronounced as ALS advances. (C) The percentage distribution of cortical excitability states, based on normative data (hyperexcitable, normal, and hypoexcitable), is illustrated across ALS phenotypes. The results show that hyperexcitability is significantly more prevalent in LMN‐dominant phenotypes, such as LMN and flail, whereas hypoexcitability is more frequently observed in UMN‐dominant phenotypes, including pyramidal and PLS (χ^2^ < 0.001). (D) the percentage distribution of cortical excitability states is shown across the number of body regions involved (1–3). The data indicate that hyperexcitability is most common in early stages (King's stage 1) but diminishes as the disease progresses. Conversely, hypoexcitability becomes increasingly frequent in later stages (King's Stage 3, χ^2^ < 0.001). In (A,C), the symbols ^, #, § and ° denote statistically significant differences between the PLS and pyramidal groups compared with the LMN, flail, bulbar, and classic groups. In (B,D), *p < 0.05, **p < 0.01, ***p < 0.001, and ****p < 0.0001. [Color figure can be viewed at www.annalsofneurology.org]

The analysis of the MEP:CMAP ratio in the upper limb across different clinical phenotypes revealed a clear gradient of UMN involvement (Kruskal–Wallis test, *p* < 0.001; Fig 3A). The LMN phenotype showed the highest MEP:CMAP ratios, whereas the pyramidal and PLS phenotypes showed the lowest, reflecting hypoexcitable corticospinal pathways and significant UMN degeneration. Classic, bulbar, and flail phenotypes showed intermediate values, representing a continuum of mixed UMN and LMN involvement.

Post‐hoc pairwise comparisons revealed significant differences between the LMN, classic, bulbar, and flail phenotypes when compared with both the pyramidal (adjusted *p* = 0.002, <0.0001, <0.0001, and <0.0001, respectively) and PLS phenotypes (adjusted *p* < 0.0001 for all comparisons). No significant difference was observed between PLS and pyramidal phenotypes (*p* = 0.47). Detailed results are shown in Fig 3A and Table S1. We also grouped phenotypes by predominant motor neuron involvement: pure‐predominant LMN (ie, LMN and flail), mixed (ie, classic and bulbar), and pure‐predominant UMN (ie, PLS and pyramidal). This analysis confirmed significant differences among the 3 groups (Kruskal–Wallis test, *p* < 0.001), with the pure‐predominant LMN group showing higher MEP:CMAP ratios than both mixed (adjusted *p* = 0.011) and pure‐predominant UMN groups (adjusted *p* < 0.0001). These findings are shown in Figure S1.

In healthy controls, the MEP:CMAP ratio had a median value of 0.33 (IQR 0.24–0.42). No significant differences were found between healthy controls and the ALS group across all hand muscles (*p* = 0.74) or within the ALS subgroup for the first dorsal interosseous muscle (*p* = 0.32).

Based on the thresholds derived from the 25th and 75th percentiles of the healthy controls, ALS patients with a ratio <0.237 (25th percentile) were classified as hypoexcitable, those with a ratio between 0.237 and 0.419 (75th percentile) were considered to have normal excitability, and those with a ratio >0.419 were defined as hyperexcitable.

The distribution of the excitability states varied significantly across ALS clinical phenotypes (χ^2^ = 72.37, *p* < 0.0001; Fig 3C). Patients with the LMN phenotype showed a predominance of hyperexcitable cases (71.4%), consistent with the heightened motor neuron excitability that characterizes this phenotype. Conversely, the pyramidal and PLS phenotypes were dominated by hypoexcitable cases (65.9% and 78.8%, respectively), reflecting marked corticospinal tract dysfunction and degeneration of the UMNs. The flail (arm and leg), classic, and bulbar phenotypes showed a more balanced distribution of excitability states, indicative of their mixed upper and lower motor neuron involvement. Post‐hoc pairwise comparisons revealed significant differences between the LMN, classic, bulbar, and flail phenotypes when compared with both the pyramidal (adjusted *p* < 0.001, 0.005, 0.01, and <0.001, respectively) and PLS phenotypes (adjusted *p* < 0.001 for all comparisons).

When stratified by King's clinical staging system, the absolute value of the MEP:CMAP ratio showed a significant decline with advancing disease stages (*p* < 0.0001; Fig 3B). Patients in stage 1 showed the highest ratios, suggesting that in the early stages of ALS, mechanisms of UMN hyperexcitability may dominate. Patients in stage 2 showed intermediate values, marking a transition from hyperexcitability‐driven mechanisms to those dominated by axonal degeneration. Post‐hoc analyses identified significant differences between stage 1 and Stage 3 (adjusted *p* < 0.001), and between stage 2 and Stage 3 (adjusted *p* < 0.01). Detailed results are shown in Fig 3B.

Similarly, stratification of the MEP:CMAP ratio based on normative data by King's clinical stages revealed a significant association between excitability states and disease stage (χ^2^ = 19.98, *p* < 0.0001; Fig 3D). Hyperexcitable cases were more frequent in stage 1, whereas hypoexcitable cases predominated in Stage 3. Stage 2 showed a transitional pattern between these 2 states. Post‐hoc analyses confirmed significant differences between stage 1 and Stage 3 (adjusted *p* < 0.001), and between stage 2 and Stage 3 (adjusted *p* = 0.036; Fig 3D).

Additional clinical data stratified based on excitability profiles revealed that age was significantly different across the groups (Kruskal–Wallis test, *p* = 0.001). Post‐hoc analysis showed that patients in the hypoexcitable group were significantly younger than those in the normal and hyperexcitable groups (all *p* < 0.013). A marked difference was observed in PUMNS scores (Kruskal–Wallis test, *p* < 0.001), specifically, the hypoexcitable group had significantly higher scores than both the normal excitable (*p* < 0.017) and hyperexcitable groups (*p* < 0.001). Additionally, the normal excitable group had higher scores than the hyperexcitable group (*p* = 0.022). All the other parameters did not show any statistical differences across the 3 groups (Table 2).

**TABLE 2:** Clinical Characteristics of Amyotrophic Lateral Sclerosis Patients Across Cortical Excitability States

	Hypoexcitable (n = 220)	Normal excitable (n = 90)	Hyperexcitable (n = 200)	*p* value	Total (N = 510)
Median age, yr (IQR)	60.0 (53.0–69.0)	68.0 (58.5–73)	66.0 (57–73)	**0.001** ^a^	63.0 (55.0–72.0)
Male count, n (%)	129 (58.6%)	57 (63.3%)	123 (61.5%)	0.703^b^	309 (60.6%)
Median disease duration at time of testing, months (IQR)	14.0 (8.0–24.0)	11.0 (6.0–25.0)	12.0 (8–21.75)	0.154^a^	13.0 (7–24)
Median ALSFRS‐R, total score (IQR)	39.0 (34.0–43.0)	41.0 (37.0–44.0)	40.0 (34–43.75)	0.105^a^	40.0 (34–43)
Median rate of progression, pts/month (IQR)	0.61 (0.28–1.15)	0.66 (0.3–1.05)	0.63 (0.33–1.07)	0.993^a^	0.63 (0.33–1.10)
Bulbar onset, n (%)	49 (22.3%)	23 (25.6%)	42 (21%)	0.453^b^	114 (22.4%)
Riluzole, n (%)	158 (71.8%)	66 (73.3%)	137 (68.5%)	0.523^b^	361 (70.8%)
Median PUMNS, total score (IQR)	13.0 (7.0–19.0)	10.0 (4.75–16)	8.0 (3.25–12.75)	**<0.001** ^a^	10.0 (5–16)
Genetic testing, n positive/total tested (%)	16/59 (27.1%)	7/13 (53.8%)	19/55 (34.5%)	0.171^b^	42/127 (33.1%)

Values are expressed as median (interquartile range) or as percentage (frequencies). Values in bold type indicate significance *p* < 0.05.

^a^ Comparisons Kruskal–Wallis test.

^b^ χ^2^‐test.

Abbreviations: ALSFRS‐R = Amyotrophic Lateral Sclerosis Functional Rating Scale‐Revised; IQR = interquartile range; PUMNS = Penn upper motor neuron score.

### Survival Impact of Cortical Excitability Profiles

**FIGURE 4:**
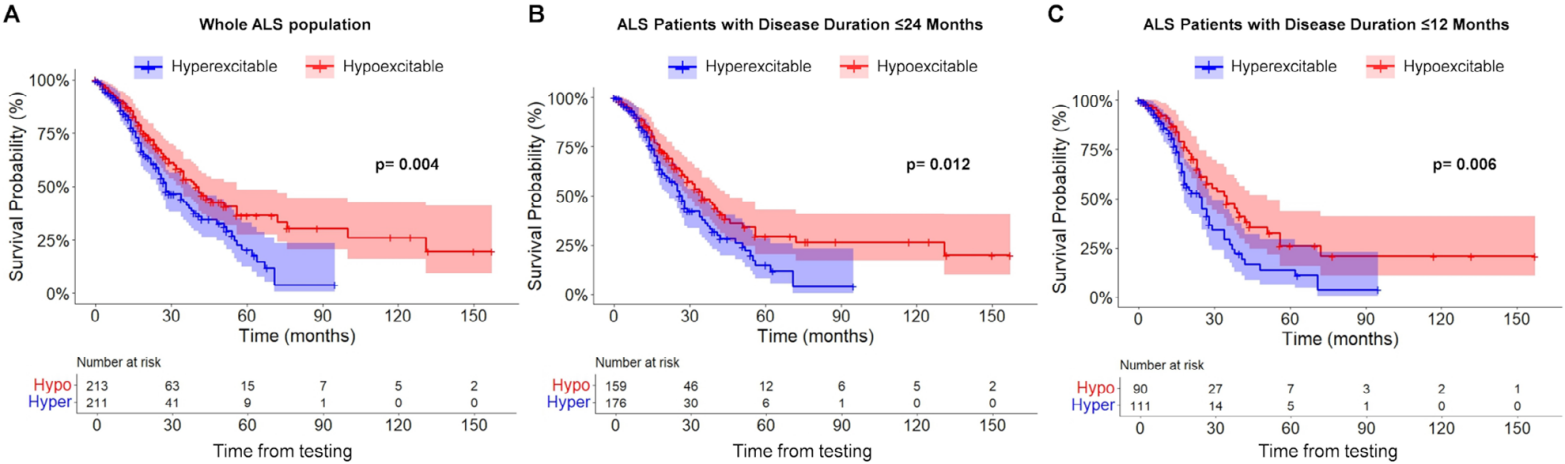
Kaplan–Meier survival analysis of amyotrophic lateral sclerosis (ALS) patients stratified by cortical excitability. (A) Kaplan–Meier survival curves for the entire ALS cohort, stratified by cortical excitability status. Patients classified as hyperexcitable (blue curve), defined by a motor evoked potential/compound muscle action potential (MEP:CMAP) amplitude ratio exceeding the 75th percentile of normative values, showed significantly reduced survival compared with hypoexcitable patients (red curve), whose ratios were below the 25th percentile (*p* = 0.004). (B) A subgroup analysis of patients with disease duration ≤24 months at the time of the transcranial magnetic stimulation assessment (*n* = 335). Hyperexcitability remained significantly associated with decreased survival (*p* = 0.012). (C) The analysis was further restricted to patients with disease duration ≤12 months (*n* = 201), reaffirming the association between hyperexcitability and poorer survival outcomes (*p* = 0.006). Collectively, these findings underscore the prognostic relevance of cortical excitability, particularly in the early stages of ALS. [Color figure can be viewed at www.annalsofneurology.org]

Kaplan–Meier survival analysis (Fig 4A) demonstrated that hyperexcitable patients (*n* = 211) had significantly shorter survival compared with hypoexcitable patients (*n* = 213; log‐rank test, *p* = 0.004). The independent prognostic value of the MEP:CMAP ratio was confirmed in a Cox proportional hazards model, where it remained a significant predictor of survival (HR 1.84, 95% CI 1.12–3.03, *p* = 0.016) after adjusting for clinical and TMS covariates (Table 3). Recognizing that hyperexcitability has been previously associated with reduced survival in the early stages of ALS,^12^ we also conducted subgroup analyses focusing on ALS patients with disease duration at the time of TMS testing ≤24 and ≤12 months (Fig 4B, C). These analyses demonstrated continued statistical significance, with hyperexcitable patients showing worse survival in both groups (≤24 months, *p* = 0.012; ≤12 months, *p* = 0.006). To further support the robustness of our findings, we split the full sample into pseudo‐randomly assigned discovery and confirmation cohorts. The prognostic value of the MEP:CMAP ratio was replicated in both subgroups, with statistically significant results in each (see Figures S2 and S3).

**TABLE 3:** Multivariate Cox Analysis of Prognostic Factors for Overall Survival

Variable	HR (95% CI)/χ^2^	*p*
Age (per yr)	1.03 (1.01–1.04)	**<0.001**
Onset site (ref: spinal)	χ^2^ = 8.56	**0.014**
Bulbar onset	1.91 (0.26–13.83)	0.411
Unknown	4.060 (0.55–30.14)	0.171
ALSFRS‐R rate (points lost/mo)	1.13 (1.02–1.25)	**0.018**
Phenotype group (ref: PLS)	χ^2^ = 24.31	**<0.001**
Classic	5.19 (2.31–11.66)	**<0.001**
Bulbar	4.87 (1.78–13.34)	**0.002**
Pyramidal	1.17 (0.14–9.78)	0.887
LMN	2.6 (0.98–6.9)	0.056
Flail	3.14 (1.10–6.49)	**0.029**
MEP:CMAP ratio	1.84 (1.12–3.03)	**0.016**

All variables shown were retained in the final multivariable model. The following variables were tested, but excluded in the final model due to lack of statistical significance: sex, riluzole therapy (yes/no), PUMNS total score, CMCT value. For multilevel categorical variables (onset site, phenotype group), overall significance is reported using the likelihood‐ratio χ^2^ statistic. Values in bold indicate statistically significant results (*p* < 0.05).

Abbreviations: ALSFRS‐R = Amyotrophic Lateral Sclerosis Functional Rating Scale‐Revised; CI = confidence interval; CMAP = compound muscle action potential; HR = hazard ratio; MEP = motor evoked potential.

These findings underscore the robust prognostic utility of hyperexcitability across different contexts, particularly in the early stages of ALS.

## Discussion

This study provides new insights into the continuum of cortical excitability in ALS, based on routinely available testing of corticospinal excitability. It highlights the transition from hyperexcitability in early disease stages to hypoexcitability and cortical inexcitability in advanced stages, demonstrating significant prognostic implications. Hyperexcitability, assessed via the MEP:CMAP ratio, was associated with shorter survival and aggressive disease progression. Conversely, hypoexcitability and cortical inexcitability correlated with advanced corticospinal degeneration and higher upper motor neuron burden. These findings underscore the heterogeneity of ALS and the utility of corticospinal excitability markers in stratifying patients across phenotype and disease stage.

### Cortical Excitability as a Function of Clinical Staging

In the early stages of the disease, cortical hyperexcitability, characterized by increased MEP:CMAP ratio, predominates. This heightened excitability is primarily attributed to an imbalance between excitatory and inhibitory mechanisms within the motor cortex, specifically reduced GABAergic inhibition and excessive glutamatergic activity.^3^, ^6^, ^33^ These neurophysiological changes appear to be an early hallmark of ALS and are closely linked to disease aggressiveness, as cortical hyperexcitability has been associated with shorter survival times.^12^ This finding aligns with previous studies indicating that reduced short‐interval intracortical inhibition (SICI) is associated with poorer prognosis in ALS, correlating with faster disease progression and worse clinical outcomes, particularly in patients with shorter disease duration.^12^ However, as our study does not directly assess SICI, we cannot determine whether cortical hyperexcitability arises from a specific deficit in intracortical inhibition. Furthermore, SICI is influenced by multiple factors related to overall cortical excitability, and its reduction does not necessarily imply a selective impairment of the GABAergic system.^34^, ^35^

Here, we confirm the findings of Shibuya et al.,^12^ demonstrating that cortical hyperexcitability, assessed through a routine neurophysiological parameter, serves as a strong independent predictor of survival in ALS patients. Importantly, this result was valid across the entire ALS phenotype spectrum, further reinforcing the critical role of cortical dysfunction in disease progression and prognosis.

As the disease advances, the state of cortical hyperexcitability gradually transitions to hypoexcitability and, ultimately, to cortical inexcitability, reflecting the progressive degeneration of corticospinal pathways. In the later stages, cortical inexcitability becomes prominent, mirroring the widespread loss of functional cortical motor neurons and their connections, which leads to the failure to generate MEPs, with higher motor thresholds, despite relatively preserved peripheral motor function.^15^ This progressive decline in cortical excitability parallels increasing UMN burden and advancing clinical staging.^12^, ^15^

These findings contrast with those reported by Menon et al.,^36^ who observed a progressive increase in cortical excitability across King's stages and in a small longitudinal ALS cohort. However, their study excluded approximately 13% of patients with high motor thresholds or cortical inexcitability, thereby selecting only individuals with preserved corticospinal function. Therefore, although Menon et al.^36^ captured a subgroup in which cortical excitability increases over time, our inclusion of the full clinical spectrum, including patients with marked hypoexcitability, likely accounts for the declining excitability observed in our cohort. Together, these complementary perspectives reinforce the concept that cortical hyperexcitability is an early and dynamic feature of ALS, which may decline as disease progresses, and cortical output deteriorates.

The evolving excitability profile closely mirrors disease progression, underscoring the utility of neurophysiological measures as stage‐specific biomarkers, with potential implications for clinical trials and prognosis. In this context, reduction of SICI has been proposed as a highly sensitive and specific biomarker for ALS diagnosis.^22^ However, the application of threshold‐tracking TMS requires specialized equipment and operator expertise, limiting its scalability in routine clinical settings. In contrast, the MEP:CMAP ratio represents a more accessible and pragmatic alternative, well suited for real‐world clinical settings and large‐scale stratification.

### Phenotypic Variability in Cortical Excitability

The study highlights significant variability in cortical excitability across ALS phenotypes, reflecting distinct underlying pathophysiological mechanisms. Patients with the selective or predominant LMN impairment (ie, LMN and flail phenotype) show relatively higher MEP:CMAP ratios and a greater percentage of hyperexcitability values, despite the clinical predominance of LMN signs. This suggests that UMN involvement, albeit subtle, is present even in LMN‐predominant forms, with values comparable to the classic ALS phenotypes, as previously reported.^13^, ^37^ In line with our findings, Tankisi et al.^20^ also demonstrated that cortical hyperexcitability, assessed through reduced SICI, was most pronounced in patients with minimal clinical UMN signs, using threshold‐tracking TMS. These results further support the notion that hyperexcitability is a hallmark of LMN‐dominant phenotypes, consistent with our observations based on the MEP:CMAP ratio.

Cortical hyperexcitability in the flail and LMN phenotypes challenges the assumption of exclusive LMN dysfunction, and supports the hypothesis that early motor cortical abnormalities may be a common feature across ALS phenotypes. Whether these abnormalities are an intrinsic component of ALS pathology or reflect compensatory mechanisms in response to LMN degeneration remains to be determined. Conversely, patients with pyramidal and PLS phenotypes show marked cortical hypoexcitability, which is closely associated with structural degeneration, including significant atrophy of the primary motor cortex and depletion of Betz cells in cortical layer V, as confirmed by neuroimaging and neuropathological studies.^38^, ^39^ In addition to hypoexcitability, previous studies have also reported elevated motor thresholds in PLS, reflecting profound dysfunction of corticospinal pathways and a greater degree of impairment of excitatory pathways compared with inhibitory circuits.^39^, ^40^ Unlike electromyography, TMS is not currently incorporated into the diagnostic criteria for assessing UMN integrity.^16^, ^17^ Even in the most recent consensus criteria for PLS, neurophysiological biomarkers providing laboratory evidence of UMN dysfunction are still pending validation, although the potential of TMS to quantify UMN involvement has been recognized.^24^

However, its integration could provide significant added value, particularly in extreme phenotypes, such as LMN‐dominant and flail variants, by revealing clinically silent UMN involvement. Furthermore, in phenotypes such as PLS, characterized by cortical hypoexcitability, TMS could serve as an objective tool to confirm the distinct neurophysiological profile of this variant, which is associated with a comparatively better prognosis.^25^

### Clinical and Therapeutic Implications of the MEP:CMAP Ratio

The MEP:CMAP ratio offers a practical biomarker for stratifying ALS patients across the full spectrum of cortical excitability, from hyperexcitability to hypoexcitability and inexcitability. This classification provides critical prognostic information and may guide therapeutic strategies. In early disease stages, hyperexcitability likely reflects a compensatory response to motor neuron loss and has been associated with poorer survival.^12^ Targeted interventions aimed at reducing excitotoxicity, such as anti‐glutamatergic therapies like riluzole, may thus be most effective in this subgroup. Conversely, hypoexcitability emerges in advanced stages as corticospinal pathway degenerate, suggesting that approaches aimed at enhancing cortical output could be more appropriate. Neuromodulatory interventions, including non‐invasive brain stimulation, may also benefit from this stratification framework.^41^ High‐frequency TMS or anodal transcranial direct current stimulation protocols might be suited for hypoexcitable patients, whereas inhibitory paradigms, such as low‐frequency TMS or cathodal transcranial direct current stimulation, may be indicated in hyperexcitable individuals.^6^, ^9^ By integrating excitability profiling into therapeutic decision‐making, the MEP:CMAP ratio could support more personalized treatment approaches and improve patient selection in clinical trials.^41^

### Limitations and Future Directions

Although this study leverages a large, multicenter cohort, certain limitations and strengths warrant consideration. The cross‐sectional design precludes direct observation of longitudinal changes in excitability. However, the inclusion of data from multiple neurophysiological centers, despite methodological differences, strengthens this real‐world study by demonstrating the robustness of findings across phenotypes, disease stages, and survival outcomes. Future studies incorporating longitudinal neurophysiological assessments and standardized protocols together with neuroimaging data will be essential to further enhance the reproducibility and generalizability of present results.

## Conclusion

In conclusion, this real‐world study elucidates the continuum of cortical excitability in ALS, highlighting its prognostic significance and phenotypic variability. The findings emphasize the utility of the MEP:CMAP ratio as a practical biomarker for stratifying patients and guiding therapeutic interventions. By advancing our understanding of cortical excitability dynamics, this work paves the way for more personalized approaches to ALS management.

## AUTHOR CONTRIBUTIONS

**Federico Ranieri:** Conceptualization; methodology; software; data curation; formal analysis; writing – review and editing; validation; visualization. **Gianmaria Senerchia:** Conceptualization; methodology; software; writing – review and editing. **Luigi Bonan:** Investigation. **Stefania Casali:** Investigation. **Corrado Cabona:** Investigation. **Mariagiovanna Cantone:** Investigation. **Fabiola De Marchi:** Investigation. **Luca Diamanti:** Investigation. **Alberto Doretti:** Investigation. **Nicola Fini:** Investigation. **Massimiliano Filosto:** Investigation. **Andrea Fortuna:** Investigation. **Aniello Iovino:** Investigation. **Valentina Virginia Iuzzolino:** Investigation. **Giuseppe Lanza:** Investigation. **Christian Lunetta:** Investigation. **Luca Maderna:** Investigation. **Jessica Mandrioli:** Investigation. **Letizia Mazzini:** Investigation. **Gabriella Musumeci:** Investigation. **Andi Nuredini:** Investigation. **Gianni Sorarù:** Investigation. **Antonella Toriello:** Investigation. **Nicola Ticozzi:** Investigation. **Massimiliano Todisco:** Investigation. **Veria Vacchiano:** Investigation. **Lucia Zinno:** Investigation. **Vincenzo Silani:** Investigation; writing – review and editing. **Simone Rossi:** Investigation; visualization; writing – review and editing; supervision; conceptualization. **Vincenzo Di Lazzaro:** Supervision; visualization; writing – review and editing; investigation; conceptualization. **Raffaele Dubbioso:** Conceptualization; investigation; writing – original draft; writing – review and editing; supervision; data curation; software; formal analysis; methodology; validation.

## Potential Conflicts of Interest

Nothing to report.

## Study Group Collaborators

The following members of the MND Study Group of the Italian Neurological Society (SIN) and the Neurostimulation‐Neuromodulation Study Group of the Italian Society of Clinical Neurophysiology (SINC) contributed to patient recruitment and data collection, and are listed in accordance with journal guidelines. In addition, members of the MND Study Group of the Italian Neurological Society (SIN) and the Neurostimulation‐Neuromodulation Study Group of the Italian Society of Clinical Neurophysiology (SINC) contributed to patient recruitment and data collection (see Study Group collaborators below).

MND Study Group of the Italian Neurological Society (SIN).

Francesca Calvi, Veneto Regional Center for Motor Neuron Diseases, Department of Neurosciences, University Hospital of Padova, Padova, Italy.

Maria Caputo, University of Modena and Reggio Emilia, Azienda Ospedaliero Universitaria di Modena, Modena, Italy.

Stefano Gazzina, Unit of Neurology, ASST Spedali Civili, Brescia, Italy.

Laura Filippi, IRCCS Ospedale Policlinico San Martino, Division of Clinical Neurophysiology, Genova, Italy.

Rachele Piras, Neurorehabilitation Department, Istituti Clinici Scientifici Maugeri IRCCS, Milan, Italy.

Marilisa Boscarino, Neurorehabilitation Department, Istituti Clinici Scientifici Maugeri IRCCS, Milan, Italy.

Rita Bella, Department of Medical and Surgical Sciences and Advanced Technologies “G. F. Ingrassia”, University of Catania, Catania, Italy.

Manuela Pennisi, Department of Biomedical and Biotechnological Sciences, University of Catania, Catania, Italy.

Neurostimulation‐Neuromodulation Study Group of the Italian Society of Clinical Neurophysiology (SINC).

Fabio Pilato, Department of Medicine and Surgery, Unit of Neurology, Neurophysiology, Neurobiology and Psychiatry, Università Campus Bio‐Medico di Roma, Rome, Italy.

Rosa Iodice, Department of Neurosciences, Reproductive Sciences and Odontostomatology, University of Naples Federico II, Naples, Italy.

## Supporting information


Table S1.
**Figure S1**.
**Figure S2**.
**Figure S3**.

## Data Availability

The data supporting the findings of this study are available from the corresponding author upon reasonable request.
