# Supplementary material for: Cortical Excitability as a Prognostic and Phenotypic Stratification Biomarker in Amyotrophic Lateral Sclerosis
Source: Ann Neurol. 2025 Jun 25;98(4):801–13. doi: 10.1002/ana.27305 (PMC12542322; doi:10.1002/ana.27305)
Supplement: Supplementary file 1 — Table S1. Figure S1. Figure S2. Figure S3. [file ANA-98-801-s001.docx]

# **Materials and methods**

**Neurophysiological assessment**

All neurophysiological procedures were performed according to standard clinical and research TMS guidelines (Groppa et al., 2012; Rossini et al., 2015; Vucic et al., 2023). TMS was delivered using a 90-mm circular coil connected to three types of magnetic stimulators: Magstim (n = 480), Ates STM9000 (n = 173), and MagPro (n = 90). MEPs were recorded from the FDI, thenar (abductor pollicis brevis or opponens pollicis), and ADM muscles, and the most affected or dominant side was selected based on CMCT criteria. Suprathreshold TMS stimuli (100% MSO) were delivered to obtain at least five MEPs, and the highest peak-to-peak amplitude in millivolts was recorded. During testing, patients were instructed to perform a slight voluntary contraction of the target muscle.

The CMAP amplitude was recorded by supramaximal peripheral stimulation. The MEP/CMAP ratio was calculated and capped at 1 in cases of volume conduction artifacts. CMCT was measured by subtracting peripheral conduction time (obtained through root stimulation at C7 level) from total MEP latency. Based on CMCT, patients were classified into normal (N), pathologically prolonged (P), non-evocable cortical response (NEC), and non-evocable peripheral response (NEP), using laboratory-specific normative values (cutoff ≥ 2 SD above mean).

To define cortical excitability categories, data from 50 healthy controls (25 males and 25 females; median age 61 years, IQR 56–68) were used. TMS was delivered to the dominant-side FDI muscle using a Magstim 200 stimulator. Based on the MEP/CMAP distribution in controls, hypoexcitability was defined as values below the 25th percentile, normal excitability between the 25th and 75th, and hyperexcitability above the 75th percentile.

**References**

- Groppa S, Oliviero A, Eisen A, et al. A practical guide to diagnostic transcranial magnetic stimulation: Report of an IFCN committee. Clinical Neurophysiology 2012;123(5):858–882.
- Rossini PM, Burke D, Chen R, et al. Non-invasive electrical and magnetic stimulation of the brain, spinal cord, roots and peripheral nerves: Basic principles and procedures for routine clinical and research application. An updated report from an I.F.C.N. Committee. Clinical Neurophysiology 2015;126(6):1071–1107.
- Vucic S, Stanley Chen K-H, Kiernan MC, et al. Clinical diagnostic utility of transcranial magnetic stimulation in neurological disorders. Updated report of an IFCN committee. Clinical Neurophysiology 2023;150:131–175.

**Supplementary Table S1**

**Pairwise comparisons of ALS phenotypes for the upper limb MEP/CMAP neurophysiological parameter**

| **Phenotype 1** | **Phenotype 2** | **p-value** | **Adjusted p-value** |
| --- | --- | --- | --- |
| PLS | pyramidal | 0,47 | 1 |
| PLS | classic | <0.0001 | **<0.0001** |
| PLS | bulbar | <0.0001 | **<0.0001** |
| PLS | flail | <0.0001 | **<0.0001** |
| PLS | LMN | <0.0001 | **<0.0001** |
| pyramidal | classic | <0.0001 | **<0.0001** |
| pyramidal | bulbar | <0.0001 | **<0.0001** |
| pyramidal | flail | <0.0001 | **<0.0001** |
| pyramidal | LMN | <0.0001 | **0,002** |
| classic | bulbar | 0,264 | 1 |
| classic | flail | 0,005 | 0,074 |
| classic | LMN | 0,12 | 1 |
| bulbar | flail | 0,141 | 1 |
| bulbar | LMN | 0,341 | 1 |
| flail | LMN | 0,951 | 1 |

This table reports the pairwise comparisons between clinical phenotypes of amyotrophic lateral sclerosis (ALS) for the MEP/cMAP neurophysiological parameter recorded from the upper limb muscles. Comparisons were performed using the Mann-Whitney U test following a significant Kruskal-Wallis result. Both raw p-values (“p-value”) and Bonferroni-corrected p-values (“Adjusted p-value”) are shown. Bold values indicate statistically significant differences after correction for multiple comparisons.

**Supplementary Figure S1**

**Cortical excitability across ALS phenotypes grouped by clinical motor neuron involvement**

Boxplot showing the distribution of MEP/CMAP ratios across ALS patients grouped by the clinical predominance of motor neuron involvement: pure/predominant LMN (i.e., LMN and flail phenotypes), mixed UMN and LMN (i.e., classic and bulbar phenotypes), and pure/predominant UMN (i.e., PLS and pyramidal phenotypes).
Significant differences were observed between all groups (Kruskal-Wallis χ² = 85.64, *P* < 0.001), with the pure/predominant LMN group showing the highest MEP/CMAP values.
Horizontal bars indicate statistically significant pairwise differences (****** *P* < 0.01; ******** *P* < 0.0001).

**Discovery and Confirmation Cohorts**

To assess the robustness and reproducibility of our findings, we performed an internal validation by dividing the full cohort into two pseudo-independent subgroups: a discovery cohort and a confirmation cohort. The split was performed in a pseudorandom fashion, with approximately 70% of the patients assigned to the discovery cohort and the remaining 30% to the confirmation cohort. This allocation was stratified to preserve the distribution of key demographic and clinical variables.

Patients were classified into Hypoexcitability and Hyperexcitability groups based on distribution-derived cut-offs of the MEP/CMAP ratio, corresponding to the 25th and 75th percentiles, respectively. Intermediate values (normal excitability group) were excluded from this specific analysis to enhance contrast between groups.

The resulting sample sizes were as follows:

- Discovery cohort: 357 patients total, including 153 in the Hypoexcitability group and 144 in the Hyperexcitability group.
- Confirmation cohort: 154 patients total, including 60 in the Hypoexcitability group and 67 in the Hypoexcitability group.

Kaplan–Meier survival analysis was performed separately in each cohort.

In the discovery cohort, the Hypo group demonstrated significantly longer survival than the Hyper group (median survival: 41 vs 35 months), with a statistically significant difference (Log-Rank χ² = 4.211, p = 0.040). These results are shown in Supplementary Figure S2.

In the confirmation cohort, the survival difference was even more pronounced (median survival: 39 vs 22 months), with strong statistical significance (Log-Rank χ² = 7.805, p = 0.005). These findings are illustrated in Supplementary Figure S3.

This two-step approach supports the reproducibility of the prognostic value of the MEP/CMAP ratio in independently derived patient subsets, despite the real-world clinical heterogeneity of the cohort.

**Supplementary Figure S2**

**Kaplan–Meier survival curves in the discovery cohort stratified by motor cortical excitability.**


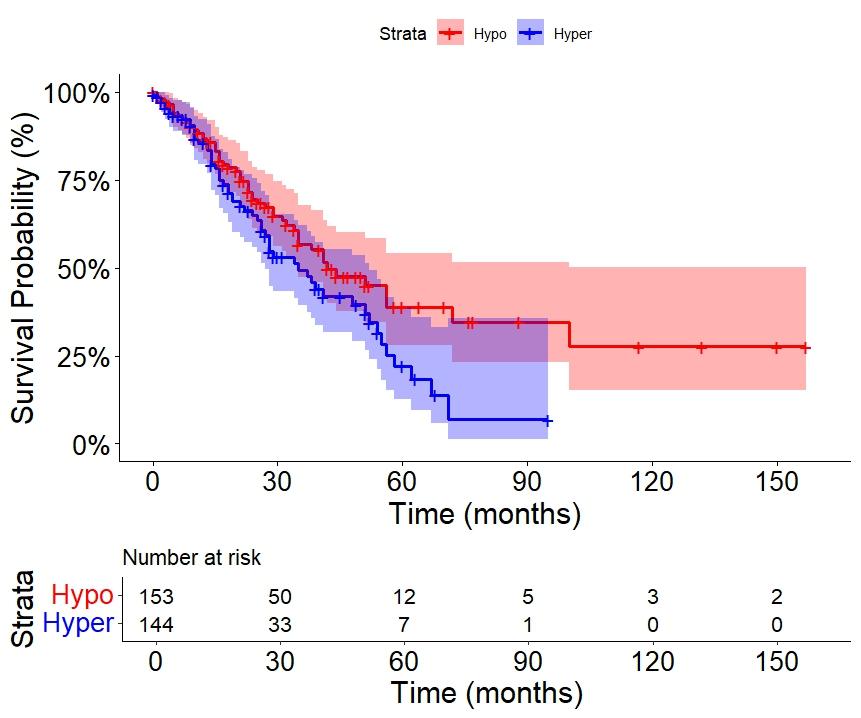


**p= 0.04**

Patients were categorized into Hypoexcitability (n = 153) and Hyperexcitability (n = 144) groups based on percentile-derived thresholds of the upper limb MEP/CMAP ratio. The Hypo group showed a significantly longer survival compared to the Hyper group (median survival: 41 vs 35 months; Log-Rank χ² = 4.211, p = 0.040). Shaded areas represent 95% confidence intervals. The number of patients at risk over time is displayed below the survival plot.

**Supplementary Figure S3**

**Kaplan–Meier survival curves in the confirmation cohort stratified by motor cortical excitability.**


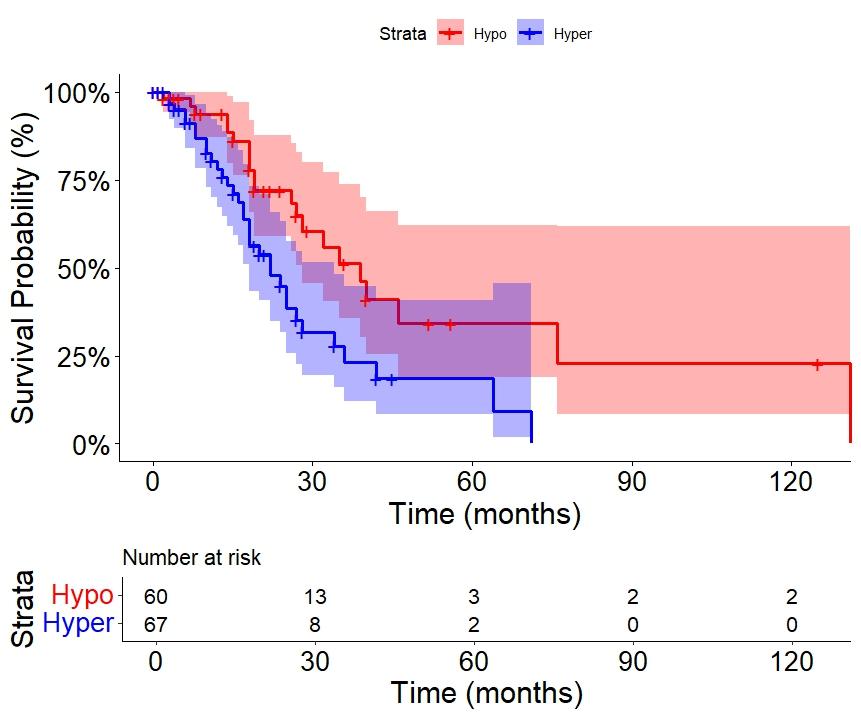


Patients were classified into Hypoexcitability (n = 60) and Hyperexcitability (n = 67) groups based on percentile-derived thresholds of the upper limb MEP/CMAP ratio. A significant survival difference was observed between groups, with the Hypo group showing longer survival (median survival: 39 vs 22 months; Log-Rank χ² = 7.805, p = 0.005). Shaded areas represent 95% confidence intervals. The number of patients at risk over time is shown below the survival plot.
